# Plant Cellular and Molecular Biotechnology: Following Mariotti’s Steps

**DOI:** 10.3390/plants8010018

**Published:** 2019-01-10

**Authors:** Angelo De Paolis, Giovanna Frugis, Donato Giannino, Maria Adelaide Iannelli, Giovanni Mele, Eddo Rugini, Cristian Silvestri, Francesca Sparvoli, Giulio Testone, Maria Luisa Mauro, Chiara Nicolodi, Sofia Caretto

**Affiliations:** 1 Istituto di Scienze delle Produzioni Alimentari (ISPA), Consiglio Nazionale delle Ricerche (CNR), Via Monteroni, 73100 Lecce, Italy; angelo.depaolis@ispa.cnr.it; 2 Istituto di Biologia e Biotecnologia Agraria (IBBA), UOS Roma, Consiglio Nazionale delle Ricerche (CNR), Via Salaria Km. 29,300, Monterotondo Scalo, 00015 Roma, Italy; giovanna.frugis@cnr.it (G.F.); donato.giannino@cnr.it (D.G.); mariaadelaide.iannelli@cnr.it (M.A.I.); giovanni.mele@cnr.it (G.M.); giulio.testone@cnr.it (G.T.); chiara.nicolodi@cnr.it (C.N.); 3 Dipartimento di Scienze Agrarie e Forestali (DAFNE), Università degli Studi della Tuscia, Via San Camillo De Lellis S.N.C., 01100 Viterbo, Italy; rugini@unitus.it (E.R.); silvestri.c@unitus.it (C.S.); 4 Istituto di Biologia e Biotecnologia Agraria (IBBA), Consiglio Nazionale delle Ricerche (CNR), Via Bassini 15, 20133 Milano, Italy; sparvoli@ibba.cnr.it; 5 Dipartimento di Biologia e Biotecnologie, Sapienza Università di Roma, P.le A. Moro 5, 00185 Roma, Italy; marialuisa.mauro@uniroma1.it

**Keywords:** Plant in vitro cultures, somatic cell selection, hairy roots, *rol* genes, *Agrobacterium rhizogenes*, genetic transformation, recalcitrant species, KNOX transcription factors, plant development, tree phase change

## Abstract

This review is dedicated to the memory of Prof. Domenico Mariotti, who significantly contributed to establishing the Italian research community in Agricultural Genetics and carried out the first experiments of *Agrobacterium*-mediated plant genetic transformation and regeneration in Italy during the 1980s. Following his scientific interests as guiding principles, this review summarizes the recent advances obtained in plant biotechnology and fundamental research aiming to: (i) Exploit in vitro plant cell and tissue cultures to induce genetic variability and to produce useful metabolites; (ii) gain new insights into the biochemical function of *Agrobacterium rhizogenes rol* genes and their application to metabolite production, fruit tree transformation, and reverse genetics; (iii) improve genetic transformation in legume species, most of them recalcitrant to regeneration; (iv) untangle the potential of KNOTTED1-like homeobox (KNOX) transcription factors in plant morphogenesis as key regulators of hormonal homeostasis; and (v) elucidate the molecular mechanisms of the transition from juvenility to the adult phase in *Prunus* tree species.

## 1. Introduction

**Figure 1:**
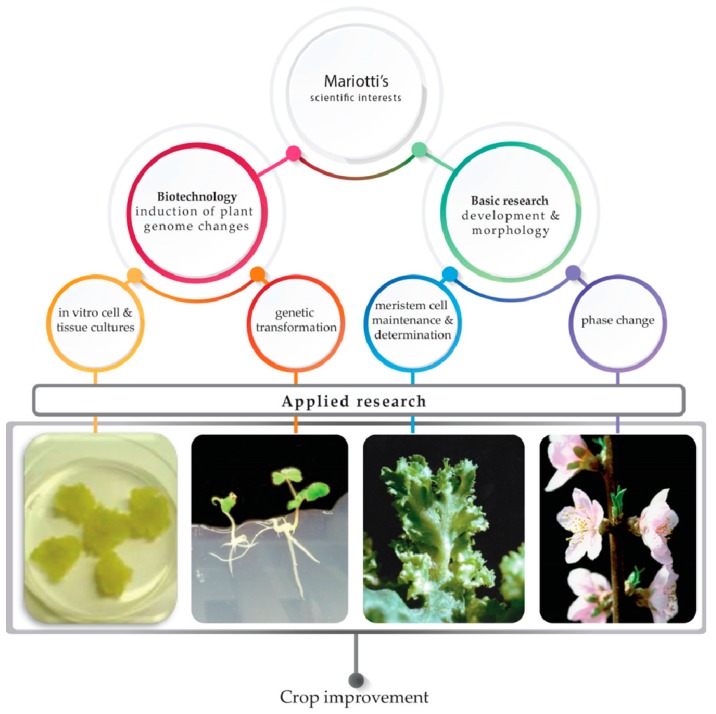
Outline of the main fields explored in this review following Mariotti’s scientific interests. His research spanned from basic research to applied biotechnology, foreseeing the great potential of in vitro cell and tissue culture for plant transformation and crop genetic improvement. All photographs in the figure have been taken by the authors of the paper.

In the 1990’s, plant biotechnology experienced a remarkable development, exerting a significant impact on genetics for crop improvement in agricultural sciences. The scientific interests of Domenico Mariotti were very much influenced by this trend, focusing on in vitro plant cell and tissue cultures of important crop species, as valuable starting tools for genetic improvement, by selecting or inducing plant genome changes. This promising scientific approach let him foresee significant achievements for applied research, as well as the possibility to add relevant new knowledge to the molecular mechanisms of plant cell development. This review, dedicated to his memory, reports on the research progress accomplished in the last 10 years, following the scientific lines drawn by his many contributions to the field of cellular and molecular biotechnology in plants of agricultural interest. His biotechnological approach will be highlighted, starting from the induction of new in vitro variability and identification of useful genetic traits for applied research (Figure 1). The study of “hairy root” syndrome induced by *Agrobacterium rhizogenes* will then be considered, in terms of new insights in the function of *rol* genes and their biotechnological application for plant genetic transformation. A specific focus regards the progress in the genetic transformation of tree species and recalcitrant legume species. As for plant development, the last two paragraphs focus on the advances on KNOX transcription factors as key regulators of hormonal homeostasis in morphogenesis, and on the study of the transition from juvenility to the adult phase in fruit trees of the *Prunus* species.

## 2. In Vitro Plant Cell and Tissue Cultures for Applied Biotechnology

In the last decades, based on the totipotency of most plant cells, many achievements have been accomplished by exploiting plant cell and tissue cultures of either model or crop species. One great potential for plant biotechnology is due to the genetic variability detectable in plant in vitro tissues, known as ‘somaclonal variation’ [1]. The exposure of plant cells to stressful in vitro conditions can enhance natural variability, which can be exploited for identifying novel useful variants. A proper selection strategy can help in identifying specific traits. To this regard, Mariotti’s group contributed to gain insight into herbicide resistance in crop species achieved by somatic cell selection, being one of the successful applications of plant biotechnology as an alternative to gene transfer. On the other hand, the use of transgenic plants has encountered several regulatory restrictions in many countries. A stepwise selection, by applying increasing concentrations of herbicide, led to the identification of carrot cell lines as resistant to the sulfonylurea herbicide, chlorsulfuron (CS). Such resistance was due to gene amplification of the target enzyme, acetohydroxyacid synthase (AHAS) [2]. Alternatively, one-step selection, by applying a single toxic concentration of the herbicide, led to the isolation of mutant forms of the AHAS enzyme in resistant tobacco and sugarbeet cells [3,4,5]. In several cases, the resistance was maintained in the plants regenerated from the resistant cell lines [6]. Since then, herbicide resistance in crops for better weed management has been widely accomplished by genetically modified plants. In particular, in the United States, glyphosate resistant crop species have been largely developed and cultivated [7]. Nevertheless, somatic cell selection has continued to be applied for crop improvement. Very recently, two variants of potato cell cultures and regenerated plants resistant to CS were identified by somatic cell selection and the resistance in both cases was due to mutant AHAS genes, confirming the effectiveness of crop cell selection for this purpose. Moreover, the identified mutant genes can be useful as selectable marker genes in potato transformation [8].

**Figure 2:**
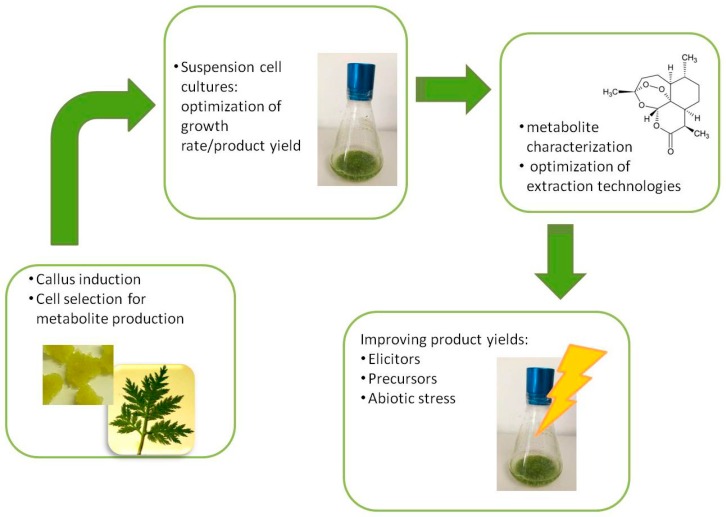
Schematic framework for the production of bioactive compounds by plant cell cultures.

The potential of in vitro variability of plant cell cultures can be of wide interest in many fields of applied research. Recently, plant cell cultures have been investigated as sources of metabolites, which can be used as food additives, pharmaceuticals, cosmetic ingredients, and as an alternative to the extraction of metabolites from field grown plants. To obtain an efficient plant cell culture process for metabolite production, it is necessary to establish cell lines by optimizing growth rate/product yields and enhancing the desired products using elicitors, precursors, or abiotic stress (Figure 2). Plant metabolite production by cell cultures can offer the advantage of a continuous supply, independent of environmental and seasonal changes, and using small spaces; moreover, it often ensures the obtainment of natural compounds that can hardly be produced in the same quality or specificity by chemical synthesis [9].

Vitamin E from plant sources comprises two groups of important antioxidant molecules, tocopherols and tocotrienols, that are differently distributed in the plant tissues [10]. The major natural vitamin E form is α-tocopherol, which can be extracted from the tissues of several food plant species [11]. Synthetic α-tocopherol, being a racemic mixture of eight different stereoisomers, is always less effective than the natural form, (R,R,R) α-tocopherol. For this reason, it is important to obtain vitamin E from natural sources, such as in vitro cell and tissue cultures [11]. Cell cultures of two oil plants, safflower and sunflower, were successfully established, producing the natural α-form as the main tocopherol [12,13]. Moreover, the sunflower in the in vitro production system confirmed that a certain degree of variability, often characterizing plant cell cultures, could be useful to identify highly productive cell lines. Two sunflower cell lines were identified and characterized for producing different amounts of α-tocopherol in cell suspension cultures’ screening. In spite of the different content of α-tocopherol (almost threefold higher in the high producing cell line, HT, than in the low producing one, LT), these cell lines had very similar growth curves. It is interesting to note that HT cells also produced higher levels of vitamin C and glutathione. On the other hand, LT cells had higher activities of antioxidant enzymes, such as ascorbate peroxidase and catalase, compared to HT [14]. Recently, suspension cell cultures of mung bean were shown to be valuable for an in vitro system for producing both antioxidant tocopherols and phytosterols [15].

Besides antioxidants, many phytochemicals belonging to the class of secondary metabolites are known to exert biological activities, which can be beneficial for human health and are of pharmaceutical interest. Human demand for these compounds has been growing along with the preference for natural products. Plant cell cultures for the production of these bioactive compounds can have significant advantages as supply sources, mainly when the desired compounds occur in very small amounts and/or are accumulated in specific tissues of the plant [16]. The apocarotenoid crocin is a main component of the yellow spice, saffron, known as a precious food ingredient with valuable pharmaceutical properties and found only in the stigma of *Crocus sativus* L. flowers [17,18]. Efforts have been made to establish crocin in in vitro production systems as an alternative to production from saffron plants, which is expensive and time-consuming. Although the induction of saffron callus cultures from stigma is very difficult to achieve, callus cultures induced from style explants were established and revealed to be more efficient in terms of the growth rate and crocin production compared to corm-derived calli, when the plant growth regulator, thidiazuron, was used [19].

As for pharmaceuticals, a successful example of efficient in vitro systems is represented by the anticancer drug, taxol, produced by cell suspension cultures of *Taxus* spp. The drug is intensively used for the treatment of different types of cancer and the cell culture technology avoids sacrificing yew trees. Such an in vitro production process has been extensively investigated and this has led to significant yield improvements. The availability of plant cell suspension cultures acting as “bio-factories” of specific compounds offers the possibility of scaling up to large volumes for industrial production. This is the case of *Taxus* cell cultures, nowadays used for industrial-scale biotechnological production to the commercialization of the anticancer drug, paclitaxel (taxol) [20].

Another plant metabolite of pharmaceutical interest is the sesquiterpene, artemisinin. It is an antimalarial compound, produced at low levels by the aerial parts, leaves, and inflorescences, of the plant, *Artemisia annua* L., an annual herb native to Asia. Due to its efficacy, it is strongly recommended by the World Health Organization as the first choice in therapeutic protocols against malaria, but unfortunately the concentration in field grown plants is quite low, being 0.1–1% dry weight, thus its worldwide supply is insufficient. Although many efforts have been made to obtain new *A. annua* genotypes characterized by enhanced yields through breeding strategies, a certain degree of variability in field grown plants was also observed [21,22]. Metabolic engineering was applied using transgenic plants of both *Artemisia* and tobacco; however, the obtained content increases of artemisinin or its precursors were not sufficient to overcome the drug shortage [23,24]. In addition, an engineered microbial system was established, however, it led to the production of the precursor, artemisinic acid, to be chemically converted to artemisinin [25]. Due to the complexity of the artemisinin molecule, chemical synthesis requires a laborious and costly process. Furthermore, it was reported that pure artemisinin was less effective than intact dried leaves in treating malaria [26], thus there is the need to explore other supply sources, such as in vitro cell culture technologies. *A. annua* in vitro cell cultures were established by optimizing the use of plant growth regulators and culture conditions. Different strategies were applied to improve artemisinin production, such as the elicitation by methyl jasmonate, which was successful for improving yields in both suspension cell cultures and hairy root cultures of *A. annua* [27,28]. The availability of suspension cell cultures has the advantage of scaling up for possible industrial production. Interestingly, *A. annua* suspension cell cultures were characterized by the ability to exudate artemisinin into the culture medium, making it easier to recover the desired native product [27]. Recently, cyclic oligosaccharides have been used in different cell culture systems for enhancing metabolite production. Resveratrol from grape cell cultures was reported to be increased by the application of β-cyclodextrins (β-CD), which acted as true elicitors [29]. Moreover, artemisinin production was significantly improved by applying different types of CD to *A. annua* cell cultures. In particular, dimethylated β-CD induced a 300-fold increase of artemisinin, most likely by reducing the negative feedback as a consequence of artemisinin-CD complex formation [30].

## 3. The “Hairy Root” Syndrome Induced by *Agrobacterium rhizogenes*

**Figure 3:**
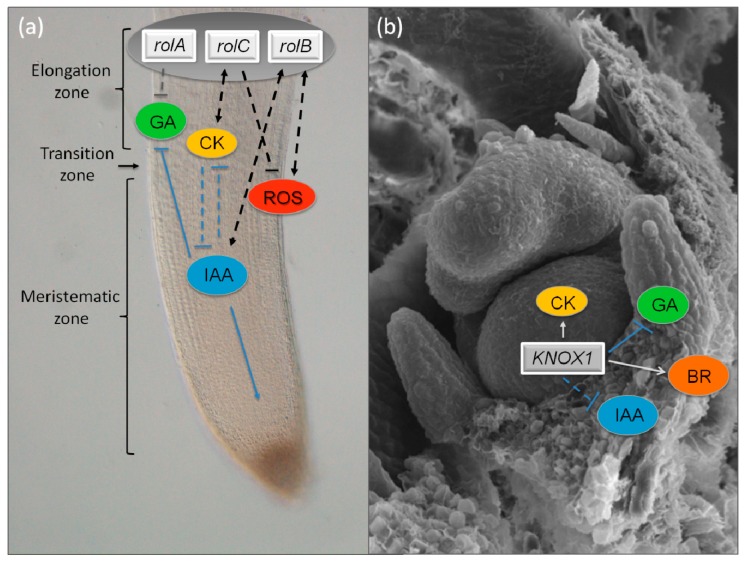
A simplified view of the involvement of *A. rhizogenes rol* genes and plant class 1 KNOX transcription factors in hormonal homeostasis in the root (left panel) or shoot (right panel) apical meristem. (**a**) *rolA, rolB*, and *rolC* may control hairy roots formation and their indefinite growth by hijacking some as-of-yet unknown components of the gibberellin (GA), auxin (IAA), and cytokinin (CK) metabolism, respectively; (**b**) class 1 KNOX control boundaries between undifferentiated cells and differentiating organs through the regulation of hormone metabolism and signaling. KNOX expression in the shoot apical meristem establishes a regime of high CK, low GA, and a gradient of auxin and brassinosteroids (BR) to keep the indeterminacy of the SAM and setting boundaries for proper organ separation during plant development.

The “hairy root” syndrome, characterized by the emergence of adventitious roots at the wound site of infected plants, was first described in the 1930s–1960s as an indicator of pathogen attack in horticultural plants. The responsible bacterial agent, *Agrobacterium rhizogenes*, was identified and the role of gene transfer from the resident bacterial plasmid to the plant genome was revealed [31]. *A. rhizogenes*, as the related *Agrobacterium tumefaciens* species, are well known for the capacity to transfer part of their DNA (Ri, root-inducing; Ti, tumor-inducing) to the plant genome during a natural infection process, leading to abnormal roots (hairy roots) or tumors (crown galls), respectively [32,33]. The expression of transfer DNA (T-DNA) causes abnormal growth and leads to the production of characteristic amino acid and sugar derivatives (opines), which can be used by the bacteria for their own growth. Being natural plant genetic engineers, in the 1980s, *A. tumefaciens* started to be exploited in biotechnology for plant genetic transformation [34]. Modified Ti plasmids, which lacked T-DNA genes related to the syndrome (disarmed), though retaining the entire *vir* (virulence) region, were used for the introduction and integration of foreign DNA in the plant cells and subsequent regeneration of transgenic plants. *A. rhizogenes* raised additional interest as Ri T-DNA transformed roots could be regenerated into whole plants with a characteristic “hairy root” phenotype. Hairy root plants have reduced apical dominance, shortened internodes, wrinkled and wider leaves, adventitious root formation, altered flower morphology, and reduced content of pollen and seeds [35], indicating a role of the T-DNA genes in modulating various developmental processes. The major *A. rhizogenes* genes involved in the hairy root syndrome were identified in 1985 among the 18 open reading frames in the T-DNA [36], and named *rol* genes (*A*, *B*, *C*, and *D*) after “rooting locus” or oncogenes for their capacity to alter plant cell programs [37]. The laboratory of Domenico Mariotti contributed to the characterization of the *rol* genes’ function [32,38,39,40], although most work was addressed to *rol* genes’ applications to induce adventitious root formation in recalcitrant species for micropropagation, and to modify developmental traits in crops [41,42,43,44,45,46]. Studies from several independent laboratories have contributed to suggest biochemical functions for the different *rol* genes [47]. The phenotype of plants transformed with either *rolA*, *rolB*, or *rolC*, and biochemical in vitro assays suggested their involvement in phytohormone homeostasis, such as gibberellins, auxin, and cytokinin metabolism and/or signaling, respectively (Figure 3a). However, conflicting results were produced, from which no definitive conclusions can be drawn. Contradictory indications were also published on the involvement of *rol* genes in reactive oxygen species (ROS) homeostasis, heading to a possible function of *rolB* in either increasing or decreasing ROS signaling [48,49], and to *rolC* as an ROS suppressor [50]. Differently, rolD was shown to act as an ornithine cyclodeaminase, which converts ornithine into proline, thus inducing acceleration and stimulation of flowering in both plants and tissue cultures [51].

Levesque et al. [52] coined the term “*plast*” genes, standing for “developmental plasticity”, to describe those *Agrobacterium* genes able to change the development when introduced into wild-type plants. According to this study, “*plast*” genes encode a family of 11 proteins (from both *A. rhizogenes* and *A. tumefaciens*), with sequence similarity values ranging between 13% and 34%, which may share similar functions, and whose diversification could result from a process of coevolution between different *Agrobacterium* species/strains and plant species. This family of ca. 70 proteins includes rolB and rolC [53] and proteins from plant species (e.g., *Nicotiana*, *Linaria*, and *Ipomoea*) that contain T-DNA genes (cellular, *cT-DNAs*) from *A. rhizogenes* in their genomes [53]. This is a very interesting example of horizontal gene transfer, which likely occurred by sparse events of spontaneous regeneration of transformed plants from *A. rhizogenes*-induced hairy roots in the natural environment. Some *Agrobacterium*-derived *cT-DNA* genes, such as *rolC*, *orf13*, and *orf14*, or some involved in opine production, are frequently intact and expressed in natural transformants, potentially able to influence plant growth and the microbiome root environment. Indeed, overexpression studies in plants suggest that “*plast*” genes have growth-modifying properties similar to their *A. rhizogenes* equivalents [54,55]. It was hypothesized that the effect of T-DNA on the regenerative capacity and the interaction with microorganism communities might have affected the evolution of natural transformant plants [56]. However, loss-of-function studies of expressed *cT-DNA* genes should be performed to assess their possible adaptive roles in plants.

Although the biochemical features of *rol* genes remain poorly understood, they have been proven to be powerful tools in plant biotechnology and functional biology research. The peculiar features displayed by hairy roots, such as a high growth rate in hormone-free liquid media, unlimited branching, and biochemical and genetic stability, make them a promising tool for metabolic engineering and large-scale metabolite production [57]. Potential applications of *rolC* and *rolD* genes in floriculture have been suggested for their effects on plant architecture and flowering promotion, respectively. Also, *rol* genes were shown to activate secondary metabolism in transformed cells from the *Solanaceae*, *Araliaceae*, *Rubiaceae*, *Vitaceae*, and *Rosaceae* families, paving the way for their possible exploitation for secondary metabolite production [57,58,59]. As an example, more than a 100-fold increase in resveratrol production was also obtained in *Vitis amurensis* cells transformed with the *rolB* bacterial gene from *A. rhizogenes* [60]. Fruits of transgenic tomato plants that overexpress *rolB* exhibited higher nutritional quality and foliar tolerance to two fungal pathogens [61], improved photosynthetic processes, and a more effective protection against oxidative damage and excess energy [62]. As *rolB* is the major activator of the secondary metabolism, its mechanism of action was further investigated, revealing a possible rolB function in activating specific MYB transcription factors to accelerate secondary metabolite production [63].

Besides biotechnological uses, an interesting application of hairy roots in fundamental biology studies exploits the ability of *A. rhizogenes* to elicit adventitious roots to obtain the so-called “composite plants”, which comprise a transgenic hairy root system attached to non-transformed shoots and leaves [64]. Initially used for micropropagation purposes, the obtainment of composite plants has become a powerful tool in gene function studies of root biology, especially those involving legume-*rhizobium* symbiosis [65]. The T-DNA harboring the transgene of interest in a disarmed binary vector is generally used to co-transform *A. rhizogenes* containing the complete Ri T-DNA, the latter allowing fast growth of transgenic roots. For these studies, relatively low virulence *A. rhizogenes* strains, such as Arqua-1 and K599, are used, which elicit a limited number of transformed roots, with growth and morphology comparable to normal roots. Transformation of *Medicago truncatula* with *A. rhizogenes* Arqua-1 allows the production of composite plants with transgenic roots that are suitable for studies of root-specific interactions because they can be nodulated by *Sinorhizobium meliloti*, efficiently colonised by endomycorrhizal fungi, and infected by pathogenic/parasitic organisms [65]. *A. rhizogenes*-transformed composite plants were achieved in different plant genera (i.e., tomato, potato, poplar) [66,67,68], including those species that are usually recalcitrant to *A. tumefaciens* transformation, providing alternative solutions in gene function studies.

Despite the huge effort made over the last three decades of research, the biochemical and cellular functions of *rol* genes, with the exception of *rolD*, remain elusive. Due to the coevolution process that occurred between *A. rhizogenes* and dicot species, *rol* genes have typical eukaryotic *cis*-regulatory motives in their promoters, but likely encode proteins of bacterial origins. Proteins encoded by *rol* genes do not display any clear sequence homology with known plant or bacterial proteins, but different and contrasting enzymatic properties have been attributed without further confirmation. Additional research to solve this “enigma” should consider that *rol* genes evolved to highjack somatic plant cells to induce root meristem initiation and maintain indeterminate adventitious root growth independently of the aerial part of the plant. Hence, the possible targets of *rol* genes should be searched amongst the main pathways involved in these root biology processes. In the past decade, most aspects of root patterning and function have been extensively explored, and the role of auxin, cytokinin, and gibberellin in root development were assessed [69], although several biochemical steps of hormone homeostasis are still unclear. Proteins encoded by *rolB* and *rolC* may be involved in as-of-yet unknown enzymatic reactions in the metabolism/signaling of these hormones in the root. This may occur either directly via already existing plant biochemical functions, or indirectly through interference with specific substrate availability, thus shifting the biochemical equilibrium. The root system of *Arabidopsis thaliana* has been established as a powerful tool to study genetic networks and signaling underlying root development [70]. It would be very interesting to study the effect of *rol* genes in the *Arabidopsis* system in light of the current knowledge on root meristem formation and maintenance. This would allow identification of candidate target genes and pathways regulated by *rol* genes at the cellular level. Moreover, the availability of complete genome information of both plants and agrobacteria, including Ri and Ti plasmids [71,72], and the possibility to run transcriptome analysis of plant-*Agrobacterium* interactions may help to integrate previous knowledge with novel molecular data to unravel *rol* genes’ mechanism of action.

## 4. Application of *A. rhizogenes rol* Genes to Fruit Tree Transformation

In the early 1980s, the *Agrobacterium rhizogenes* wt was used in fruit trees to improve propagation of difficult-to-root varieties and rootstocks. At that time, gene transfer represented a pioneeristic work in woody plants because regeneration methods were poorly available or not developed yet, considering the usual recalcitrance of these species to in vitro manipulation, as well as molecular techniques. However, after many efforts and with many initial failures, the work was rewarded with many positive results, which consisted of chimeric or fully transformed plants; the former was achieved by bacterial direct inoculum through a wound at the base of the shoot, while the latter was produced by whole plant regeneration (shoot organogenesis or somatic embryogenesis) from “hairy roots”. Later, transgenic whole plants were obtained for one or few *rol* genes of the *riT-DNA* plasmid of *Agrobacterium tumefaciens*. Several traits of fruit species were successfully modified by genes of *A. rhizogenes* and the major results are summarized in Table 1. The first woody plants modified with *A. rhizogenes* NCPPB pRi1855, using in vitro micro-shoots, were almond cv Tuono [73] and, later, olive cv Moraiolo [74,75]. Both species showed abundant rooting in auxin free medium or in very low auxin concentration, while in almond, the detached roots continued to grow in vitro even in hormone-free medium and to produce opines, and those of olive plants rarely expressed these abilities. The reason could be ascribed to transient gene expression or to the organogenesis of non-transformed cells, after stimuli from the adjacent transgenic ones or the bacterium diffusible exudates [76]. Olive plants showed less vigor than those rooted with auxin, similarly to plum MrS2/5, cherry F12/1, and cherry rootstocks Colt in field conditions [77]. Subsequently, the *A. rhizogenes* gene transfer technology to induce in vitro rooting spread throughout several fruit species (Table 1).

While many species are easily induced to in vitro rooting by *A. rhizogenes* wt, in vivo experiments proved difficult or impossible. Rinallo and Mariotti [45], after unsuccessful experiments with *A. rhizogenes* wt, obtained abundant rooting in chestnut cuttings using *A. tumefaciens* harboring the *rolB* gene, in combination with etiolation and auxin treatments. Later, it has been demonstrated that auxins and putrescine play an important co-adjuvant role in *A. rhizogenes*-mediated root induction [75]. Only cuttings from seedlings of *Asimina triloba* L. were responsive to *A. rhizogenes* treatment; therefore, juvenility should be considered a key factor for successful transformation [90]. According to Sutter and Luza [91], plant response to *A. rhizogenes* involves auxins through either hormone increased concentration or increased sensitivity of the infected cells, based on the analogies of the morphological response of plant tissues treated with auxins.

Whole transformed plants with *riT-DNA* were achieved following the regeneration from “hairy roots” in papaya [81], cherry rootstock Colt [92], and kiwifruit [93], which showed the typical hairy root syndrome. Plant regeneration of fully transgenic plants is feasible in vitro and in vivo (in the pot or in the field) from spontaneous regeneration of hairy roots, particularly in species (e.g., *Prunus* spp.) that show high efficiency of regeneration from roots [79]. However, the “hairy root” phenotype is exhibited not only by fully transformed plants, but also by chimeric plants (having only transformed roots). This phenomenon limits the use of *A. rhizogenes* wt to overcome the difficulties encountered in the rooting of hard-to-root species, since sole transgenic roots also modify the canopy morphology. Nonetheless, a large scale selection of *Prunus* spp. regenerated form hairy cultures was effective to produce *riT-DNA* dwarfing rootstocks that did not alter the fruit quality of grafted conventional sweet cherry scions [77]. These novel approaches have the advantage of shortening the time required for selection and escape the stringent regulations on genetically modified organisms, because no recombinant vector is used. The idea of producing *riT-DNA* transgenic plants with a high rooting ability of (mature) cuttings is still challenging as seen in *riT-DNA* Colt rootstocks, which showed rooting recalcitrance by hardwood and semi-hardwood cuttings, and also by layering in the field [77,79], while the explants easily rooted in vitro, even without auxin supply.

To avoid the strong “hairy root” phenotype, *rol* genes from the *riT-DNA* were cloned into *A. tumefaciens* to produce several transgenic fruit plants. Specifically, through induced shoot organogenesis from leaves, male *rolABC* “GTH” [44,74] and female “Hayward” kiwifruits were produced [43] together with many offsprings (*rolABC* “GTH” × “Hayward” control), and, subsequently, *rolABC* “Canino” olive tree, through cyclic somatic embryogenesis of maternal tissue [84,85], and 10 years of field trials were also conducted. Overall, the transgenic *rolABC* phenotype is characterized by pleiotropic effects; they include: Internode and shoot shortening; reduction of trunk, leaf lamina, and petioles; reduced number of total flowers and increased number of single flower per bud; delay of vegetative growth in autumn; increased rooting ability in vitro and in vivo; increased tolerance to drought and decreased transpiration rate; increase of putrescine levels; enhanced *Pseudomonas syringae* susceptibility [94]; and fruit shape alteration and dwarfing properties of rootstocks [44,95]. Several of these traits also occurred in other *rolABC* transgenic fruit trees, including cherry ‘Inmil’ (*P. incisa* × *serrala*) and Damil (*P. dawyckensis*) [96] and walnut hybrid [97], whereas in transgenic *Citrus* spp. plants, a higher photosynthetic efficiency, better development of root systems, and higher tolerance to oxidative stress were reported [98]. Furthermore, the soils underneath transgenic plants did not change in its composition of microbial populations [82]. The same behavior has been observed in other species, such as *rolABC* olive cv Canino, in field trials, where the plants showed a strong reduction of apical dominance with a short internode length, with a tendency to axillary buds’ outgrowth and prolonged vegetative growth in late autumn with a high risk of frost damage in winter [83]. Regarding the single *rol* transformation, *rolB* female kiwifruit appeared morphologically similar to the controls, with a slight increase in fruit size and a normal shape; nevertheless, a reduction in the number of triple flowers per bud (the triple flowers is a negative phenomenon in the female cultivar, Hayward), a higher drought tolerance, and self-rooting were scored [77,99]. In apples, *rolB* induced the typical hairy root phenotype and transgenic rootstocks affected the internode length, canopy size, flowering, and fruiting of the conventional scion, whilst the fruit quality was preserved [86,87,100,101]. RT-PCR analysis revealed that neither the *rolB* gene nor its mRNA were detectable in the scion, indicating no translocation from the rootstock to scion. Similar results have been observed in the pear rootstock [88] and in grapes [80]. *RolC* gene insertion into kiwifruit (*A. deliciosa* A. Chev) generated yellow leaves, stunted growth, and reduction of fruit size and flower number, thus was unsuitable for commercial uses [99]. *RolC* plants have been produced also in *A. kolomikta* [102], in *Fragraria × ananassa*, cv Calipso, and raspberry [103]. In the latter species, the increase of cytokinins’ metabolism was accompanied with increased yield and fruit downsizing, enhanced sugar content and tolerance to *Phytophthora cactorum* [103], boosted rooting ability, and precocious flowering [89]. *RolC* overexpression reduced the vigor in pear rootstocks [104] and in *Poncirus trifoliatae*, together with the internode shortening, enhanced rooting ability [105].

Overall, the whole *riT-DNA* of *A. rhizogenes* and *rol* genes, singly or in association, merit further investigation, since the results so far obtained suggest a favorable use for improving different fruit tree species, both varieties and rootstocks, to be used in modern agriculture, suitable for mechanization and for adverse soil and climate conditions. In addition, the use of wild type bacterium could also allow the stringent rules of genetically modified organism regulations to be overcome.

## 5. Genetic Transformation of Legumes

In her review on “Advances in development of transgenic pulse crops” published in 2008, Susan Eapen wrote: ‘To date, genetic transformation has been reported in all the major pulse crops like *Vigna* species, *Cicer arietinum*, *Cajanus cajan*, *Phaseolus* spp., *Lupinus* spp., *Vicia* spp. and *Pisum sativum*, but transgenic pulse crops have not yet been commercially released. The reason for lack of commercialization of transgenic pulse crops can be attributed to the difficulty in developing transgenics with reproducibility, which in turn is due to lack of competent totipotent cells for transformation, long periods required for developing transgenics and lack of coordinated research efforts by the scientific community and long term funding’ [106].

One of the main interests of Domenico Mariotti was the genetic transformation of crop plants, in particular grain legumes, mediated by *Agrobacterium*. These crops are recalcitrant to in vitro culture and this makes it more difficult to achieve genetic transformation. Mariotti was very clear that the key toward success was to be able to reach the meristematic areas and then stimulate organ regeneration, avoiding the callus phase. With this in mind, he contributed to establishing protocols for chickpea and common bean transformation [107,108].

Nowadays, 10 years later, things have not gone very far. Few transgenic legume crops have been approved and registered for commercialization, most of which have been produced in soybean [109], alfalfa [110], and only one is in a common bean, the EMBRAPA EMB-PVØ51-1 variety, resistant to Bean Golden Mosaic Virus [111]; however, only GM soybean and alfalfa are currently cultivated.

Compared to other crops, progress in legume transformation is still very poor. Besides technical problems, this may be due to the lower economical relevance of some of these crops compared to cereals, despite the increasing interest that is arising for legumes in the last years, and to the fact that most of them are mainly cultivated and consumed in developing countries of Asia (*Cajanus cajan*, *Cicer arietinum*, *Lens culinaris*, *Vigna radiate*, and *Vigna mungo*), Africa (*Vigna unguiculata*, *Phaseolus vulgaris*), and Central and South America (*Phaseolus vulgaris*) [112]. Furthermore, the strict regulations imposed by several European countries on GM crops cultivation have strongly limited the economic interest as well as the technical advancements in recalcitrant crops, such as legumes. Therefore, despite the importance of pulse legumes to both human and agroecosystem health, these crop species still lack a high throughput genetic transformation system. Main limiting technical factors regard the recalcitrance of pulses for regeneration, low competency of regenerating cells for transformation, and lack of a reproducible in planta transformation system [106,113]. *Agrobacterium tumefaciens*-mediated gene transfer is still the most commonly used procedure for legume transformation. Consistent attempts for high-frequency recovery of transgenic events with *Agrobacterium*-mediated transformation in major grain legumes have resulted in marginal success, despite optimization of several crucial parameters [114,115]. Some good results have been obtained with direct gene transfer using particle gun bombardment, a technique that is mostly genotype independent and that may overcome problems related to plant regeneration [116]. In fact, legume in vitro regeneration is still a challenge for plant researchers; however, the extensive use of the model legume plants, *Medicago truncatula* and *Lotus japonicas*, for molecular studies has favored the development of efficient regeneration and *Agrobacterium*-mediated transformation protocols for these two species [114].

Root transformation using *A. rhizogenes* has emerged as an alternative to traditional transformation and is gaining importance as an effective tool for reverse genetics studies in plants, especially legumes in which studies have focused on genes involved in root biology and root–microbe interactions [114,115]. For example, transgenic adventitious roots have been proven to be a good system to investigate the role of genes involved in symbiosis [116].

In vitro regeneration of legumes is based on direct organogenesis, indirect organogenesis, or somatic embryogenesis from different explant types. The determination of species-specific parameters, like the explant source, plant genotype, and media components, are key to gain successful regeneration. When possible, the somatic embryogenesis approach is favored, as each event of regeneration is supposed to be derived from one cell and chromosomal rearrangements are less frequent, however, this system may increase the frequency of unwanted traits arising from somaclonal variation. In many cases, the regeneration of shoots from the cotyledonary node or from other meristematic explants after *Agrobacterium* infection has been proven to be a rapid and relatively efficient method in a number of legume species [113]. Mariotti’s group contributed to this field, proposing a method to obtain common bean plant regeneration from different genotypes, through meristematic organogenesis [117]. However, the pioneering work of Domenico Mariotti and co-workers started before, when in 1989, they published a first study reporting the development of transgenic common bean and runner bean (*P. vulgaris* and *P. coccineus*, respectively) plants based on a rapid and efficient plant regeneration system, which reduced the in vitro culture and avoided the callus phase [108]. The transformation method was based on *A. tumefaciens* infection of the primary node of young explants deprived of both apical meristem and the upper part of axillary buds. They obtained good percentages (15–20%) of shoot regenerations on the selective media for both species, and among these, about 60% were positive to GUS staining [114]. Unfortunately, in the paper, no data were presented on the stability through generation of the transformants, so it remains to be demonstrated that the efficacy of the method can produce stable transformed T1 and T2 plants. A few years later, Domenico Mariotti and his coworkers reported the first transformed chickpea plantlets obtained after co-cultivation of embryonic axis [107].

Subsequently, several reports were made of chickpea transformation using the embryonic axis or parts thereof. Indeed, frequent common features of legume crop transformation protocols include the use of cotyledonary nodes or embryonic axes as explants for genetic transformation, the use of grafting to overcome problems related to organogenesis, and the addition of thiols compounds to improve the transformation efficiency [114,118,119,120,121].

Although we are still far from efficient and high throughput transformation systems, for some legume crops (chickpea, cowpea, lupin, common bean, peanut), a number of successful transformation events have been reported in the last 10 years, underlying the development of robust transformation methods, although very often still poorly efficient and genotype dependent. Chickpea has been transformed for resistance against target pests, bruchids and aphids, as well as for traits conferring tolerance to drought and salinity [122]. In all these works, transgenic chickpea plants were always obtained by *Agrobacterium*-mediated methods, with only one exception, in which the method used was based on particle gun bombardment [123]. Some progress has been gained also with the transformation of *Vigna* species (*V. unguiculata*, *V. radiate*, and *V. mungo*) and transgenic plants have obtained resistance to biotic stresses, abiotic stresses, or herbicides [124,125,126,127]. Only cowpea lines tolerant to a herbicide from the imidazoline class (imazapyr) were obtained by means of particle gun bombardment [127]; in all other cases, transformation was achieved by the use of *Agrobacterium tumefaciens*. Improved protocols, based on the method set up by Pigeaire et al. [128], are also available for lupin species’ (*Lupinus angustifolius*, *L. luteus*) transformation [129,130] and have been applied to develop plants that are resistant to fungal disease [131] or to improve the seed sulphur amino acid content [132]. Common bean, the only food legume crop for which a GM variety has been approved, was transformed by the use of the biolistic method [133,134]; however, a recent paper reported the possibility to transform this crop by *Agrobacterium*-mediated transformation using indirect organogenesis [135]. Successful genetic transformation protocols have been reported in the peanut both via *Agrobacterium tumefaciens* [136,137] and biolistic/particle bombardment [138]. Moreover, several papers report examples of genetic transformation of peanuts to improve traits related to abiotic and biotic stresses and for the production of oral vaccines [139]. Very few reports are available for other legume crops, such as the lentil [140] and faba bean [141].

In the last years, the emergence of genome-editing technologies has revolutionized plant research, and it is now possible to create specific and precise genetic modification as well as modulate the function of DNA sequences in their endogenous genomic context [142]. The power of this new technology has been accompanied with a burst of edited crops to speed up breeding. In the near future, we can expect that increasing efforts will be put into advancing knowledge and technical skills to improve genetic transformation of legumes and hopefully gaps with other crops will be reduced.

## 6. KNOX Transcription Factors as Key Regulators of Hormonal Homeostasis in Plant Morphogenesis

KNOTTED1-like homeobox (KNOX) transcription factors (TF) belong to the Three Amino acid Loop Extension (TALE) ancestral superclass of homeodomain transcription factors conserved in animals, plants, and fungi [143], and are subdivided into three phylogenetic classes (class 1, 2, and M) [144]. Functional studies of class 1 *KNOX* genes in the 1990s assigned a prominent role of KNOX transcription factors in regulating cell fate determination at the shoot apical meristem (SAM) and in leaf morphogenesis and architecture [145,146,147]. However, at that time, neither direct nor indirect relationships between the expression of *KNOX* genes and the modification of plant biochemical functions were known. In the late 1990s, a few laboratories started to hypothesize that KNOX may act through modification of hormonal homeostasis, mainly cytokinins (CKs) and gibberellins (GAs) [148]. Among these, Mariotti’s laboratory first established the occurrence of a strict correlation among *KNAT1* (an *Arabidopsis* class 1 *KNOX*), overproduction of specific cytokinins in the leaves, and leaf architecture through *KNAT1* overexpression in the crop species, *Lactuca sativa* [149]. Accumulation of cytokinins in the vascular bundles at the leaf margins suggested that KNAT1 might change the determinate state of the leaves to indeterminate by increasing cytokinins’ biosynthesis [150]. This let them hypothesize a leading role of cytokinins in leaf development and morphology, and a possible role of KNOX in the regulation of cytokinin production, though the plant genes for the cytokinin biosynthesis had not been identified yet. The discovery of plant *ISOPENTENYL TRANSFERASE* genes (*IPT*s) encoding the cytokinin biosynthetic enzymes [151,152] paved the way to establish a direct regulatory link between KNOX TFs and cytokinin biosynthesis. Independent studies in model species provided molecular evidence for the positive regulation of CK biosynthesis by KNOX in the SAM through the activation of some *IPT* genes [153,154,155], and positioned cytokinins both upstream and downstream of class 1 KNOX. Further studies on compound-leafed species confirmed a major role of cytokinins in leaf architecture by regulating morphogenetic activity in leaf margins. Shani et al. elegantly demonstrated that expression of class 1 KNOXs during leaf primordia development correlated to the maintenance of an indeterminate state that would prompt the leaf to undertake morphological processes for leaflet production [156], and that CK mediates this function in the regulation of leaf shape [157].

Gibberellins homeostasis was also placed downstream of class 1 KNOX, which were shown to directly repress GA biosynthesis and up-regulate GA catabolism [158,159,160]. These and further studies identified a key role of class 1 KNOX in maintaining high levels of CK and low levels of GAs to keep the indeterminacy of the SAM and to set boundaries for proper organ separation during plant development [161].

Indications that KNOX action may also involve modulation of the auxin pathway came from genome-wide studies in maize [162]. ChIP-seq analysis showed a direct binding of the maize KNOX KN1 to auxin-related genes, including those involved in auxin signaling and transport, and some of them showed differential expression in *Kn1-N* (gain of function mutant) leaves. Moreover, KN1 can bind genes involved in the synthesis of auxin and its precursor, tryptophan, suggesting that KN1 may directly control the auxin pathway at all levels. Several genes involved in auxin biosynthesis and transport, in GA biosynthesis and in CK catabolism, signaling, and response were also identified in a recent work as modulated by the class 1 KNOX *Arabidopsis* protein, SHOOT MERISTEMLESS (STM), using STMoe and STM-RNAi time-course data and meta-analysis [163].

In addition to cytokinins, gibberellins, and auxin, class 1 KNOXs were also shown to regulate the brassinosteroids (BRs) pathway. BRs are growth-promoting phytohormones involved in diverse aspects of plant growth and development [164]. They promote differentiation through activation of a large number of genes related to cell elongation and cell wall modification [165]. In rice, a class 1 *KNOX* gene, *OSH1*, was shown to negatively regulate the BR pathway and in particular, the genes involved in the BR catabolism [166]. The regulation of the BR catabolism is evolutionarily conserved in maize and is important for SAM function and organ boundary formation in leaves [167]. Among the different functions of the BRs, the regulation of vascular bundles’ formation and lignin deposition appears to be relevant [168]. Although a direct link among class 1 *KNOX* genes, BRs and lignin deposition is still to be determined, and the *Arabidopsis KNAT1* mutant, *brevipedicellus* (*bp*), shows increased lignin deposition in the stems [169]. Lignin mislocalization and inappropriate cell differentiation in discrete regions of *bp* stems suggests a role of KNAT1 in regulating cell wall properties, particularly lignin deposition and quality, to prevent premature cell differentiation. Characterization of a *KNAT1* ortholog in *Prunus persica* tree species, *KNOPE1*, confirmed this role in preventing lignin deposition as *KNOPE1* expression was inversely correlated with that of lignin genes and lignin deposition along the peach shoot stems and was down-regulated in lignifying vascular tissues [170].

In contrast to class 1 *KNOX* genes, which are expressed primarily in meristematic tissues, class 2 *KNOX* gene expression occurs in differentiating organs [161,171,172]. The function of class 2 KNOX proteins, as well as potential connections with hormonal pathways, has long remained unknown. Recently, the *Arabidopsis KNAT3/4/5* class 2 *KNOX* genes were shown to act redundantly to promote differentiation of aerial organs, antagonistically to the action of class 1 *KNOX* genes [173]. In *Arabidopsis*, *KNAT3/4/5* loss-of-function phenotypes were reminiscent of a gain-of-function of class 1 KNOX phenotypes, and produced leaves with altered leaf margins and shape. In the compound-leafed species, *Cardamine irsuta*, a reduction or increase in class 2 KNOX activity led to an increase or decrease in leaf complexity, respectively, confirming the antagonistic relationship between class 1 and class 2 KNOX transcription factors [173]. However, no connection with specific hormonal pathways has been described so far for class 2 KNOX in leaf development.

Evidence that class 2 KNOX TFs may act through the inhibition of the cytokinin pathway, antagonistically to class 1 KNOX proteins, came from studies on the role of *KNOX* genes in legume root nodule organogenesis. Functional studies of the *Medicago truncatula KNAT3/4/5* class 2 *KNOX* genes [174] suggested that class 2 KNOX TFs regulate legume nodule development through a cytokinin regulatory module, involving a type-A cytokinin response regulator, to control nodule organ boundaries and shape like the class 2 KNOX function in leaf development [175]. It is tempting to speculate that *KNAT3/4/5-like* genes may constitute a regulatory pathway acting in shoot and aerial organ development, which are recruited for the morphogenetic process that underlies plant-rhizobia symbiosis.

Further investigations are needed to fully comprehend the role of *KNOX* genes in developmental processes underlying plant morphogenesis. Despite their pivotal roles in controlling multiple hormonal pathways, KNOX of class 1 can directly regulate key transcription factors of important developmental processes. These TFs, which are overrepresented among target genes [163], include *CUP SHAPED COTYLEDON (CUC)* transcription factors involved in the specification of the meristem-organ boundary zone, the *TEOSINTE BRANCHED1/CYCLOIDEA/PCF1 (TCP)* family of *bHLH* that also control cell differentiation, and *AINTEGUMENTA-like (AIL) AP2* transcription factors *PLETHORA (PLT) (AIL/PLT)* that regulate pluripotency and phyllotaxis. To fully comprehend regulatory networks controlled by TALEs, studies on KNOX should be reconciled and integrated with those on BEL1-like homeobox (BLH or BELL) TFs, the other subgroup of the TALE protein family, which form functional heterodimers with KNOXs. So far, it is not known if specific KNOX-BLH complexes have a different affinity for the same targets or diversified target specificity, neither if they act as transcriptional activators of repressors in different developmental contexts. Moreover, class 1, class 2, and class M interplays need further studies to untangle the proposed antagonistic function in cell differentiation, likely mediated by different hormonal pathways, including possible regulation of common targets in an opposite way.

## 7. Phase Change in Fruit Trees: Advances and Perspectives in Peach and *Prunus* Species

Plant post-embryonic development encompasses the juvenile, adult vegetative, and reproductive phases. In tree species, the end of juvenility and the first flower appearance may not coincide, implying the occurrence of an adult vegetative phase [176]; all these transitions occur gradually along the shoot so that intermediate patterns are evident [176]. The adult vegetative-reproductive switch of meristems encompasses the perception of the flowering signal (flower induction), the meristem re-organization (flower initiation), and flower organ morphogenesis (differentiation). Tree flower buds can undergo dormancy, a growth slowdown that is abandoned after response to specific environmental conditions [177]. Rejuvenation is a reversible shift of all or part of the tree from an older to a younger phase; e.g., explants from mature trees may reverse to juvenile traits, such as enhanced rooting during tissue culture [178]. The explant age is crucial for the success of in vitro technologies. Mariotti’s group conducted research to develop phase-specific markers at the morphological, histological, cytological [179], and gene expression levels using *P. persica* as a model. Specifically, they identified differentially transcribed genes putatively subtending differences in organs of juvenile, juvenile-like, and mature shoots [180,181]. Peach juvenility spans 3–5 years and is affected by proper seedling management [182]. Juvenile and adult vegetative traits can differ in leaf size, growth vigor, and photosynthetic activity. In mature plants, the one-year branch has a major role in flowering; leaf axillary meristems produce single or clustered buds bearing single flowers or shoots in multiple combinations. These processes are under the control of the shoot growth speed, node length, and expansion grade of subtending leaves [178]. Flower induction is poorly investigated in the peach; vegetative to reproductive meristem transition and flower initiation mostly occur in summer as studied in three-bud clusters (a central vegetative plus two side flower buds). During dormancy, organ development is continuous in both vegetative and flower buds [183,184]; flower bud dormancy release is regulated by chill and heat requirements, water and nutrient conditions, and hormonal equilibria [185].

Extensive research in annual and perennial model species has unraveled gene networks of phase changes, addressing functional conservation in trees [186], and providing tools to favor allele introgression and enhance micropropagation. The juvenile to adult vegetative shift is coordinated by the decreased expression of two microRNAs, *miR156* and *miR157*, which repress the protein synthesis of SQUAMOSA PROMOTER BINDING PROTEIN-LIKE (SBP/SPL) family transcription factors. These latter are upstream regulators of *APETALA1 (AP1)*, *LEAFY (LFY)*, and *FRUITFULL (FUL)*, key MADS-box transcription factors that confer floral identity to meristems. The *SPL* genes can also control vegetative organs in adulthood, providing models that explain the co-existence of the vegetative phase change and adult vegetative-reproductive changes along the tree shoot. The *miR156*/*miR172* abundance levels can mirror the leaf stage in various species; higher contents of *miR156* vs. *miR172* mark juvenility, while the opposite typifies vegetative adulthood. As for rejuvenation, in vitro culture causes the appearance of juvenile traits accompanied by high *miR156* levels [176]. Finally, the upstream regulation of *miR156*/*SPLs* module includes gibberellin-mediated stimuli, glucose levels, several biotic and abiotic cues, the biogenesis process, and epigenetic control [187]. Regarding trees, the *miR156* ectopic expression in poplars reduces the *SPL* and *miR172* expression and prolongs juvenility, confirming evolutionary conservation [188]. In apples, two *miR156* precursors and mature forms decrease during the juvenile-adult vegetative transition; the ectopic expression of pre-*miR156* in tobacco represses the endogenous *SPL* levels and triggers adventitious rooting [189,190]. Moreover, *miR156* levels are elevated in in vitro rejuvenated explants of *Prunus* spp. [191] and peach seedlings and in vitro plants showed higher levels of *miR156* and lower expression of *SPL* and *miR172* than the adult ones [192]. Finally, in a work to which Mariotti contributed, DNA methylation was shown to be lower in meristems of young/juvenile-like shoots vs. adult ones, supporting epigenetic mechanisms being associated to phase maintenance [179].

Flowering initiation involves interactions of inner and outer stimuli able to trigger the adult vegetative-reproductive transition in the shoot apical meristem (SAM) [193]. In the *Arabidopsis* annual model, the pathways responding to internal (autonomous, gibberellin, circadian clock, age, and sugar balance) and external signals (vernalization, temperature, and photoperiod) converge towards floral integrators, which can act in the SAM as floral transition promoters or repressors that cross-interact. Major promoters are SUPPRESSOR OF OVEREXPRESSION OF CONSTANS 1 (SOC1), FLOWERING LOCUS T and D (FT and FD), and AGAMOUS-LIKE24 (AGL24) that activate meristem identity factors, such as LFY, AP1, SEPALLATA3 (SEP3), and FUL, which set the irreversible transition. Repressors are necessary to modulate the floral transition by ensuring the appropriate time-space expression of flowering promoters; key actors are FLOWERING LOCUS C (FLC), SHORT VEGETATIVE PHASE (SVP), and TERMINAL FLOWER 1 (TFL1). Focusing on the FT product, it moves from leaves to SAM, where it is bound to FD, to establish meristem re-programming/flower initiation via *SOC1* triggering. As for TFL, it represses flowering by competing vs FT in FD binding. Moreover, age-related and vernalization events share the control mechanisms based on the *miR156/SPL* and *miR172/AP2*-like modules in perennial models. Finally, the *miR172/AP2* module controls the floral destiny of axillary buds [186]. The equivalence of floral integrators/meristem genes between *Arabidopsis* and fruit trees was reported in various studies [177]. Table 2 includes some key functional studies of *Prunus spp.* genes. Models from perennials have been crucial to unravel the mechanisms of seasonal flower induction of *Prunus* trees. Contextually, Mariotti and colleagues found that the message localization patterns of a maintenance DNA-methyltransferase gene differed in vegetative vs reproductive buds during flower initiation, suggesting a role of methylation in re-programming bud fates [180].

Organ identity genes guide flower piece growth and the *Arabidopsis* ABCDE model proposes five classes of activities that act alone or in combination (A: *AP1* and *AP2* specify sepals and petals; B: *AP3* and *PISTILLATA*, petals and stamens; C: *AGAMOUS*, stamen and carpels; D: *SHATTERPROOF1* and *2* and *SEEDSTICK*, ovules; E: *SEP1-4*, redundant function). These genes encode MADS-box transcription factors and peach putative orthologues have been characterized [194]. Flower differentiation and development are under miRNA specific control [195] and many peach miRNAs have been sequenced, though they are functionally undefined [196]. AGL24-like factors (peach *DAM1-6*) control seasonal dormancy in the peach evergreen mutant and integrate day-length and temperature signals to regulate endo-dormancy [194].

Modern peach breeding exploits marker-assisted selection; the facts that juvenility length is inherited [182] and that a juvenile quantitative trait loci (QTL) was found in *P. mume* offer tools to shorten unproductive stages [197]. As for maturity in *Prunus* trees, the term “flowering time”, which should properly refer to SAM adult vegetative-reproductive transition, usually measures the number of disclosed flowers (a.k.a. blooming date). The blooming date is controlled by several QTLs that are spread over eight linkage groups and affect seasonal distribution and production. Apricot, sweet cherry, and peach maintain QTL locations though peach specific ones’ reside on group 6. These QTLs were associated to flowering genes, including *LFY* and *TFL1* [198], whereas the chilling requirement and blooming date QTLs co-localization [199] support shared determinism. QTL detections and genome wide association studies have received great benefit from peach genome sequence allowing high throughput genotyping of *Prunus* spp. and the assignment of novel QTL of flowering [200].

Biotechnology approaches can shorten the vegetative stages of both scion and rootstocks [182]. Recalcitrance to the genetic transformation of peach has been a long-lasting drawback for the low tissue regeneration efficiency [178]. Cultivar-independent protocols are still necessary and, so far, there have been no transgenic peaches with modified traits. Rootstock genetic engineering was successful and effective to control scion traits [201]. Peach gene function is currently addressed by transient RNA-interference technologies [202,203] and new approaches exploit development genes to enhance in vitro regeneration [204]. Potentially, reproductive maturity can be achieved by finely tuning the *Prunus* flowering gene expression (Table 2). Namely, the *FT* gene overexpression, which causes early and continuous flowering in the plum [205], has led to “FasTrack” breeding strategies for the rapid incorporation of important traits into desired cultivars. The system uses multiple backcrosses and molecular marker selections to produce improved and non-transgenic varieties in five years [206]. The use of recombinant viral vectors (*Apple latent spherical virus*) was effective to induce precocious flowering; the *Arabidopsis FT* delivered into apple seedlings caused the endogenous *TFL1* silencing and precocious anthesis, reducing the breeding cycle to one year [207]. Other virus-based vectors were effective to silence genes in the peach [202,203], offering tools to phase shift manipulation. Finally, recent strategies of gene editing exploit the delivery of guide RNA and Cas9 protein mixture to apple protoplasts from which non-transgenic edited lines were regenerated [208], further paving the way in *Prunus* trees. As for peach micropropagation, monitoring of miRNA expression levels can be useful to assess the maturity status and regenerative/rooting potential of explants. Hence, tuning the miRNA levels by induction can be useful to control rejuvenation, embryogenesis, and somaclonal variation associated to in vitro cultivation [209].

## 8. Conclusions

Research updates of the topics, which were in the scientific interests of Domenico Mariotti, dealing with plant genetics for crop improvement in agricultural sciences, were focused on. In the last decade, following the routes of his insights, many goals were reached from plant biotechnology applications of in vitro cell cultures to the genetic transformation of relevant crops, including new knowledge on plant organogenesis of model plants, as well as phase transition in fruit tree crops. However, despite the achieved progress, further efforts are needed to shed more light on the genetic basis of key developmental processes in model and crop species. By identifying new useful genetic traits, it will be possible to further exploit the high potential of plant cells for improving crop production.

## Figures and Tables

**Table 1:** Main results in woody fruit species obtained by the use of *riT-DNA* and *rol* genes of *Agrobacterium rhizogenes*.

Species	Gene(s)	Results	Ref.
Olive, Almond, Walnut, F12/I, MrS/5, Colt, apple	*riT-DNA*	Chimeric plants (better rooting)	[73,78,79,80]
Papaya (*Carica papaya*)	*riT-DNA*	Reduced growth habit	[81]
Colt rootstock (*P. avium × P. pseudocerasus*)	*riT-DNA*	Reduced growth habit	[79]
Kiwifruit (*Actinidia deliciosa*), cv Hayward	*rolB*	bigger fruits, drought tolerance	[43,44,81]
Kiwifruit (*A. deliciosa*), cv Hayward and GTH	*rolABC*	reduced plant size, flower set, increased drought tolerance	
Citrange Troyer (*Citru sinensis × P. trifoliata*)	*rolABC*	drought tolerance	[82]
Olive (*Olea europaea* L.) cv Canino	*rolABC*	Reduced growth habit, increased drought tolerance	[83,84,85]
Apple rootstock	*rolA*	Reduced growth habit	[86]
Apple rootstock	*rolB*	Reduced growth habit	[87]
Pear (*P. communis* L.)	*rolB*	Increased rooting ability	[88]
Strawberry (*Fragraria* × *ananassa*)	*rolC*	Higher fruit set and resistance to *Phytophtora cactorum*	[89]
Pear rootstock	*rolB*	Increased rooting ability	[88]
Richter 110 (*Vitis berlandieri × V. rupestris*)	*rolB*	better rooting	[80]

**Table 2:** Ectopic expression of some flowering genes from/into *Prunus* species.

Gene ^1^	Donor	Receiver	Assay ^2^	Phenotypic Effect	Ref.
*AP1*	*Prunus avium*	*Arabidopsis thaliana*	oe	early flowering	[210]
*CO*	*Prunus persica*	*Arabidopsis thaliana*	co	flowering promotion	[211]
*FT*	*Prunus avium*	*Arabidopsis thaliana*	oe	early flowering	[212]
	*Prunus persica*	*Arabidopsis thaliana*	co	flowering promotion	[211]
	*Populus trichocarpa*	*Prunus domestica*	oe	early flowering	[205]
*MADS5*	*Prunus persica*	*Arabidopsis thaliana*	oe	early flowering	[213]
*MADS7*	*Prunus persica*	*Arabidopsis thaliana*	oe	early flowering	[213]
*SOC1*	*Prunus mume*	*Arabidopsis thaliana*	oe	early flowering	[214]
*CBF*	*Prunus persica*	*Malus domestica*	oe	cold-induced dormancy	[215]
*DAM6*	*Prunus mume*	*Populus tremula×P. tremuloides*	oe	dormancy promotion	[216]
*SVP1*	*Prunus mume*	*Arabidopsis thaliana*	oe	flowering delay	[189]
*TFL1*	*Prunus persica*	*Arabidopsis thaliana*	oe	flowering delay	[217]

^1^, *AP1*, *APETALA1*; *CO*, *CONSTANS*; *FT*, *FLOWERING LOCUS T*; *MADS5* and *MADS7*, *SEPALLATA-like*; *SOC1*, *SUPPRESSOR OF OVEREXPRESSION OF CONSTANS 1*; *CBF*, *C-REPEAT BINDING FACTOR*; *DAM6*, *DORMANCY-ASSOCIATED MADS box6*; *SVP1*, *SHORT VEGETATIVE PHASE 1*; *TFL1*, *TERMINAL FLOWER1*. ^2^, Functional assay: oe, overexpression; co, complementation.
